# A Case Series of Myocarditis Following Third (Booster) Dose of COVID-19 Vaccination: Magnetic Resonance Imaging Study

**DOI:** 10.3389/fcvm.2022.839090

**Published:** 2022-03-04

**Authors:** Arthur Shiyovich, Guy Witberg, Yaron Aviv, Ran Kornowski, Ashraf Hamdan

**Affiliations:** Department of Cardiology, Rabin Medical Center, Tel Aviv University, Tel Aviv, Israel

**Keywords:** myocarditis, BNT162b2 messenger RNA (mRNA) COVID-19 vaccination, third dose (booster), cardiac magnetic resonance imaging (CMR), COVID-19

## Abstract

**Background:**

Myocarditis has been reported following the first two doses of Pfizer-BNT162b2 messenger RNA (mRNA) COVID-19 vaccination. Administration of a third dose (booster) of the vaccine was initiated recently in Israel.

**Objective:**

The aim of this study was to describe the characteristics of patients referred for cardiac magnetic resonance (CMR) imaging with myocarditis following the booster.

**Methods:**

Patients referred for CMR imaging with a clinical diagnosis of myocarditis within 21 days following the booster, between July 13 and November 11, 2021, were analyzed.

**Results:**

Overall, 4 patients were included, 3/4 (75%) were men, and the mean age was 27 ± 10 years. The time from booster administration to the onset of symptoms was 5.75 ± 4.8 days (range 2–14). Obstructive coronary artery disease was excluded in 3 of the patients (75%). CMR was performed 34 ± 15 days (range 8-47 days) following the 3rd vaccination. The mean left ventricular ejection fraction was 61 ± 7% (range 53–71%), and regional wall motion abnormalities were present in one of the patients. Global T1 was increased in one of the patients, while focal T1 values were increased in 3 of the patients. Global T2 was increased in one of the patients, while focal T2 values were increased in all the patients. Global ECV was increased in 3 of the patients, while focal ECV was increased in all the patients. Median late gadolinium enhancement (LGE) was 4 ± 3% (range 1–9%), with the inferolateral segment as the most common location (3 of the 4 patients). All the patients met the Updated Lake Louise Criteria.

**Conclusions:**

Patient characteristics and CMR imaging findings of myocarditis following the administration of the booster vaccine are relatively mild and consistent with those observed with the first two doses. Although larger-scale prospective studies are necessary, these initial findings are somewhat reassuring.

## Introduction

Myocarditis has been reported to be a possible rare adverse event following the first or second dose of Pfizer-BNT162b2 messenger RNA (mRNA) COVID-19 vaccination (1–4). Incidence of such post-vaccine myocarditis was reported to be highest among younger males, with most cases being mild or moderate with favorable clinical outcomes (1, 4). As reported by us (5) and others (6–8), cardiovascular magnetic resonance (CMR) imaging findings of these patients were consistently mild and in line with “classical myocarditis.” Following the resurgence of COVID-19 morbidity, the Israeli Ministry of Health announced a campaign to administer the third dose (i.e., booster) of the BNT162b2 mRNA COVID-19 vaccine (Pfizer–BioNTech) to individuals who received the second dose > 5 months earlier, starting on July 13 (9). This third vaccine dose was reported to be effectively protected against severe COVID-19-related outcomes (9). Our aim in the current report was to describe the characteristics of patients referred for CMR with myocarditis following the administration of the BNT162b2 mRNA COVID-19 vaccine.

## Methods

### Study Population

This study comprised consecutive patients who are members of Clalit Health Services (CHS), and who were referred for CMR at Mor Inside Ltd. (Kfar Saba, Israel), with a clinically suspected diagnosis of myocarditis within 21 days after receiving the third dose of the Pfizer-BNT162b2 mRNA COVID-19 vaccine between July 13, 2021, and November 11, 2021. Patient-specific data were available from referral letters and electronic medical records. Patients with prior history of myocarditis, with missing data of the third dose of the vaccine, or with an alternative competing diagnosis (i.e., COVID-19 infection) were excluded.

This study was approved by the CHS institutional review board and performed consistently with the Helsinki declaration. Exemption from informed consent was granted.

### CMR Imaging

The patients underwent CMR imaging using a 1.5 T scanner (Ingenia; Philips Medical Systems). The CMR protocol included multiplanar cine imaging and late gadolinium enhancement (LGE) imaging. T1 mapping was performed using a balanced steady state free precession, single breath-hold modified inversion recovery Look-Locker (MOLLI). T2 mapping was performed using a navigator-gated black blood-prepared gradient spin-echo sequence. Native T1 and T2 mapping, and postcontrast T1 mapping were acquired in apical, mid-ventricular, and basal short-axis slices.

Data analysis was performed using a dedicated CMR workstation (Philips Intellispace Portal, version 11.0). Cardiac volume, function, and mass were measured using a semiautomated contour detection system, and extracellular volume (ECV) was calculated based on pre and postcontrast T1 images. Myocardial ROIs was placed accurately to minimize partial volume effects from adjacent blood pool or extra-myocardial tissues. Global T1 and T2 relaxation times and ECV were evaluated for the complete mid-ventricular slice using motion-corrected images as previously described (10). Consistent with Puntmann et al. (10), to avoid overestimation of T1 value due to partial volume effect, the apical slices were not analyzed. In addition, there are no differences in T1 value between basal and mid-ventricular slices (11), and in some cases, the basal slice may contain a part of the left ventricular outflow tract (11).

Regional T1, T2, and ECV were measured in LGE positive myocardium by manually drawing a region-of-interest (ROI) on the LGE image around the lesions and copying these ROIs to the corresponding T1 and T2 maps; respectively.

LGE was defined as an image intensity level ≥ 2 SDs above the mean of the remote myocardium. Abnormal native T1, T2, and ECV values were defined as >1,060 ms, >57 ms, and higher than 28%, respectively (12). The diameter of pericardial effusion was measured at the end-systolic frame, and pericardial LGE was considered present when enhancement involved parietal and visceral pericardial layers.

We evaluated the diagnosis of myocarditis by CMR using the Updated Lake Louise Criteria (13).

### Statistical Analysis

A descriptive statistical methodology was used. Patient characteristics are presented as counts (%) for categorical variables and mean (±SD) or median (range) for continuous variables.

## Results

Overall, 4 patients met the inclusion criteria. A total of ¾ th (75%) were male, and the mean age was 27 ± 10 years (range: 18–44 years). Baseline characteristics are presented in Table 1. One of the patients had asthma, but the rest were otherwise healthy. The mean time from the third vaccine administration to the onset of symptoms was 5.7 5 ± 4.8 days (range 2–14). Of all the patients who experienced chest pain, ¾th (75%) had abnormal ECG mostly accounting for ST-segment elevations, and troponin levels were increased in all the patients, with peak values between 79 and 4,967 ng/L. Obstructive coronary artery disease was excluded in 3 (75%) of the patients, one had coronary angiography, and the other two had coronary computed tomography angiography.

**Table 1:** Clinical characteristics and CMR findings of the study patients.

**Age (ye- ars)**	**Sex**	**Past medi- cal his- tory**	**Symp- toms**	**ECG**	**Peak Tro- ponin (ng/L)**	**CAD ruled out**	**Time from 3rd vaccine and symp- toms (days)**	**Time bet- ween 3rd vaccine and MRI (days)**	**LVEF %**	**Wall motion abnorm- ality**	**LVEDV/ BSA**	**LVESV/ BSA**	**LV- mass/ BSA**	**T1 global (ms)**	**T1 focal (ms)**	**T2 global (ms)**	**T2 focal (ms)**	**Global ECV (%)**	**Focal ECV (%)**	**LGE (%)**	**LGE local- ization**	**LGE pat-tern**	**LGE in peri- card**	**Peri- car- dial effu- sion**	**Dia- meter of effu- sion (mm)**
21	M	None	Chest pain	Infe- rior STE	240	CA	4	8	53	Lateral wall	73.5	33.9	49.6	1,078 ± 107	1,135 ± 118	62 ± 8	69.2	30.1	36	9	Antero- lateral, infero- lateral (basal, mid) Lateral (apical)	Epicar- dial and mid-wall	Y	N	
44	F	None	Chest pain	Nor- mal	80	CCT	2	40	63	N	70.6	25.8	31.7	1,039 ± 70	1,077 ± 66	52.4 ± 6	57.5	30.5	31.9	1	Apex, infero- septal (basal)	Mid-wall	Y	N	
26	M	As- thma	Chest pain	Diff- use STE	4,967	N	14	47	71	N	76.7	22.2	46.9	1,045 ± 93	1,155 ± 89	50 ± 6.7	58.1	34.2	44.9	3	Inferior and infero- lateral (basal)	Epicar- dial	N	Circular	5
18	M	None	Chest pain	Diff- use STE	79	CCT	3	42	59	N	74	31.4	45.8	1,008 ± 70	1,041 ± 80	49 ± 4.4	57.4	27.3	29.3	1	Inferior (basal)	Epicar- dial	N	Circular	4

*M, male; Y, yes; N, no; LV, left ventricular; LVEDV, left ventricular end-diastolic volume; ECG, electrocardiogram; LVESV, left ventricular end-systolic volume; BSA, body surface are; LGE, late gadolinium enhancement; CAD, coronary artery disease; CA, coronary angiography; CCT, cardiac computed tomography; STE, ST-segment elevation*.

**Figure 1:**
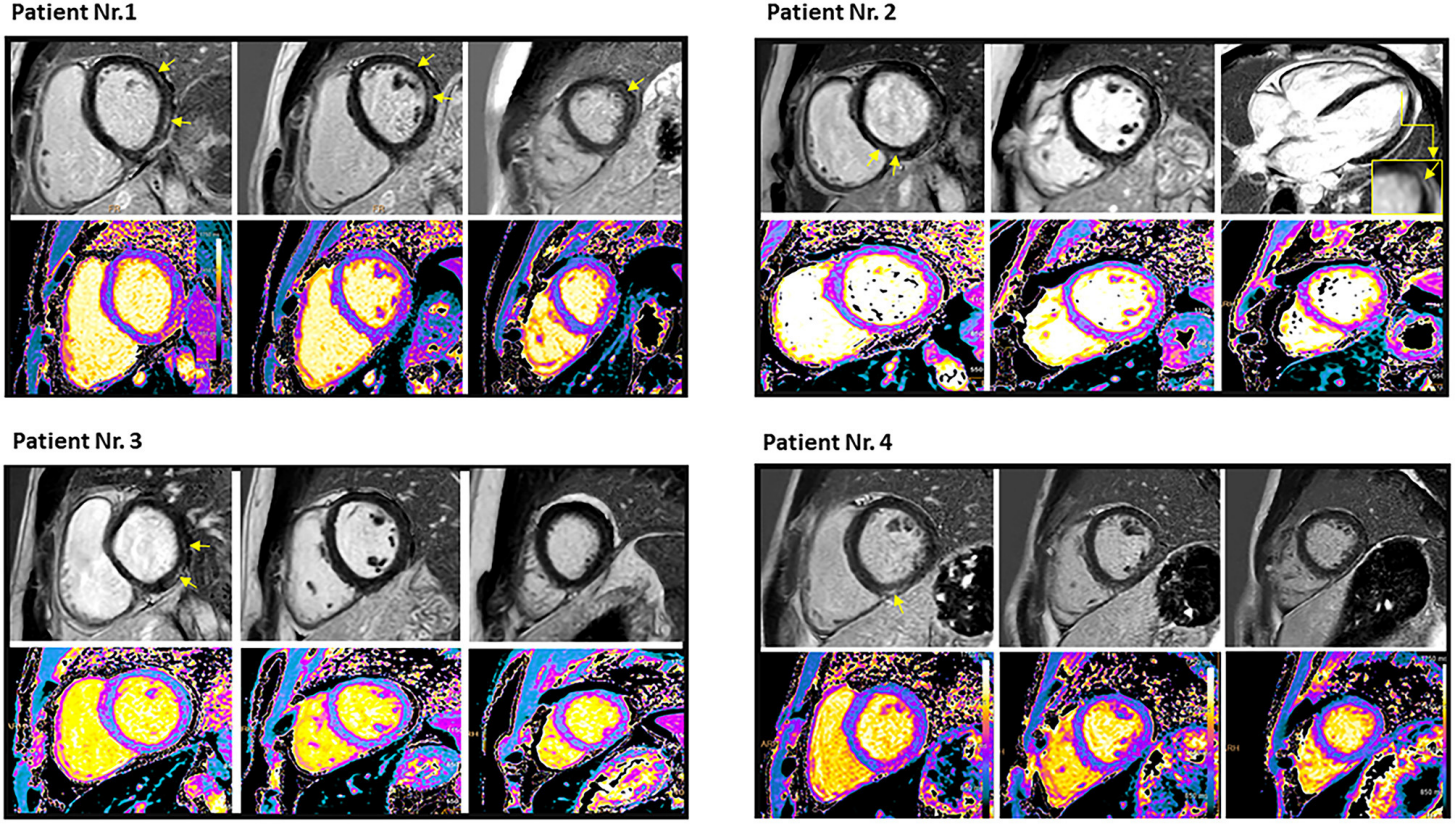
Cardiac magnetic resonance imaging of the four patients who had myocarditis following the third dose of mRNA COVID-19 vaccination demonstrated late gadolinium enhancement (yellow arrows) and T1 mapping (lower row). Patient no. 1: Mid wall late gadolinium enhancement involving 9% of the myocardium with corresponding myocardial injury in native T1 mapping imaging in antero- and infero-lateral segments of basal and mid ventricular short-axis view, as well as in the lateral segment of apical short-axis view. Native T1 value was 1,135 ms, and T2 value was 69.2 ms. Peak troponin was 240 ng/L, and scan delay (from COVID-19 vaccine) was 8 days. Patient no. 2: Mid wall late gadolinium enhancement involving 1% of the myocardium with corresponding myocardial injury in native T1 mapping imaging in the lateral segment of apical and in the septal segment of the basal short-axis view. Native T1 value was 1,077 ms, and T2 value was 57.5 ms. Peak troponin was 80 ng/L, and scan delay (from COVID-19 vaccine) was 40 days. Patient no. 3: Epicardial late gadolinium enhancement involving 3% of the myocardium with corresponding myocardial injury in native T1 mapping imaging in the inferior and inferolateral segments of the basal short-axis view. Native T1 value was 1,155 ms, and T2 value was 58.1 ms. Peak troponin T was 4,967 ng/L, and scan delay (from COVID-19 vaccine) was 47 days. Patient no. 4: Mid wall late gadolinium enhancement involving 1% of the myocardium with corresponding myocardial injury in native T1 mapping imaging in inferior segments of the basal and mid-ventricular short axis view, as well as in the lateral segment of the apical short axis view. Native T1 value was 1,041 ms, and T2 value was 57.4 ms. Peak troponin T was79 ng/L, and scan delay (from COVID-19 vaccine) was 42 days. Reference (normal) values: T1: 950–1,060 ms, T2: < 57 ms, and troponin T < 13 ng/L.

The CMR imaging was performed after a median of 34 ± 15 days (range 8-47 days) following the 3rd vaccination. One of the patients underwent CMR during the acute phase, while the rest over a month following the acute episode. The CMR findings are presented in Table 1. CMR images of all the patients are presented in Figure 1.

The mean left ventricular ejection fraction was 61 ± 7% (range 53-71%), regional wall motion abnormalities were present in one of the patients only. Global T1 values were increased in one (25%) of the patients, while focal values were increased in 3 (75%) of the patients. Global T2 values were increased in one (25%) of the patients, while focal values were increased in all of the patients (100%). Global ECV was increased in 3 (75%) of the patients, while focal ECV was increased in all the patients (100%). LGE was present in all the patients; thus, all of the patients met the Updated Lake Louise Criteria. Mean LGE% was 4 ± 3% (range 1-9%), and the inferolateral segment was the most common location (3/4 patients). LGE patterns were as follows: epicardial 2 patients, mid-wall 1 patient, mid-wall and epicardial 1 patient. LGE in the pericardium was present in 2 of the 4 patients, and pericardial effusion was present in 2 of the 4 patients, circular in both. The diameter of pericardial effusion was 4 and 5 mm in the two latter patients.

## Discussion

The present study consists, to our knowledge, of the first report describing CMR as well as clinical findings of patients with myocarditis following the administration of BNT162b2 mRNA COVID-19 booster (i.e., 3rd dose) vaccine. The baseline characteristics of the patients in this report are consistent with those of people who developed myocarditis following the first two doses, as previously reported (5–8); most were young men without a significant past medical history. However, it should be mentioned that one of the patients (25%) was a 44-year-old woman, which could imply less dominance of men with myocarditis following the 3rd vaccine, yet this is a small cohort thus such inferences are significantly limited. The CMR findings are overall mild, with two patients having ~1% LGE, and consistent with those previously reported following the first two doses of the vaccine (5–8). Although this could partially result from the delayed scan, it is probably consistent with the favorable outcome of these patients. Findings are also similar to those reported on patients who recently recovered from COVID-19, suggesting potential etiological common pathways for myocardial involvement (10). The severity of the CMR findings (e.g., LGE percentage, T1 values, etc.) was greater in one patient, in whom CMR was performed during the acute phase compared with over a month delay in the other patients. Although this may imply the natural course of the inflammation, a selection bias with a more severe case scanned earlier cannot be ruled out. Nevertheless, and despite the delay in CMR in 3 of the patients, all the patients met the Updated Lake Louise Criteria (13).

## Limitations

The causality between myocarditis and the vaccine cannot be unequivocally determined. However, temporal proximity between the two events and the very similar characteristics of the patients and previously reported CMR findings to support a probable causal association. An additional limitation is that CMR was performed over a month after the acute phase in 3 of the 4 patients, which might have attenuated some of the findings. We should also acknowledge the relatively small cohort, which limits the generalizability of the findings.

## Conclusions

Patient characteristics and CMR findings of clinically suspected myocarditis following the administration of the booster vaccine are relatively mild and consistent with previous observations following the first two doses. Although more data are required to better characterize this clinical entity, these initial findings are somewhat reassuring with regard to the risk/benefit profile of the third dose of the vaccine.

## Data Availability Statement

The raw data supporting the conclusions of this article will be made available by the authors, without undue reservation.

## Ethics Statement

The studies involving human participants were reviewed and approved by Helsiniki Committee Clalit Healthcare Services. Written informed consent for participation was not required for this study in accordance with the national legislation and the institutional requirements.

## Author Contributions

AS, AH, and RK conceived and planned the study. AS and AH reviewed the CMR tests, contributed to the interpretation of the results, and drafted the manuscript. AS, AH, YA, and GW obtained patient related clinical data and contributed to sample preparation. All authors provided critical feedback and helped shape the research, analysis, and manuscript. All authors contributed to the article and approved the submitted version.

## Conflict of Interest

The authors declare that the research was conducted in the absence of any commercial or financial relationships that could be construed as a potential conflict of interest.

## Publisher's Note

All claims expressed in this article are solely those of the authors and do not necessarily represent those of their affiliated organizations, or those of the publisher, the editors and the reviewers. Any product that may be evaluated in this article, or claim that may be made by its manufacturer, is not guaranteed or endorsed by the publisher.
